# Why Are Women Dying When They Reach Hospital on Time? A Systematic Review of the ‘Third Delay’

**DOI:** 10.1371/journal.pone.0063846

**Published:** 2013-05-21

**Authors:** Hannah E. Knight, Alice Self, Stephen H. Kennedy

**Affiliations:** 1 Nuffield Department of Obstetrics & Gynaecology, University of Oxford, Oxford, United Kingdom; 2 Oxford Maternal & Perinatal Health Institute, University of Oxford, Oxford, United Kingdom; 3 Sandwell General Hospital, Lyndon, West Bromwich, United Kingdom; University of Vermont College of Medicine, United States of America

## Abstract

**Background:**

The ‘three delays model’ attempts to explain delays in women accessing emergency obstetric care as the result of: 1) decision-making, 2) accessing services and 3) receipt of appropriate care once a health facility is reached. The third delay, although under-researched, is likely to be a source of considerable inequity in access to emergency obstetric care in developing countries. The aim of this systematic review was to identify and categorise specific facility-level barriers to the provision of evidence-based maternal health care in developing countries.

**Methods and Findings:**

Five electronic databases were systematically searched using a 4-way strategy that combined search terms related to: 1) maternal health care; 2) maternity units; 3) barriers, and 4) developing countries. Forty-three original research articles were eligible to be included in the review. Thirty-two barriers to the receipt of timely and appropriate obstetric care at the facility level were identified and categorised into six emerging themes (Drugs and equipment; Policy and guidelines; Human resources; Facility infrastructure; Patient-related and Referral-related). Two investigators independently recorded the frequency with which barriers relating to the third delay were reported in the literature. The most commonly cited barriers were inadequate training/skills mix (86%); drug procurement/logistics problems (65%); staff shortages (60%); lack of equipment (51%) and low staff motivation (44%).

**Conclusions:**

This review highlights how a focus on patient-side delays in the decision to seek care can conceal the fact that many health facilities in the developing world are still chronically under-resourced and unable to cope effectively with serious obstetric complications. We stress the importance of addressing supply-side barriers alongside demand-side factors if further reductions in maternal mortality are to be achieved.

## Introduction

The massive difference in the maternal mortality ratio (MMR) between rich and poor countries is one of the largest disparities of any public health statistic, including under-five mortality [1]. While there has been real progress in reducing mortality rates in children under five [2], the reduction in MMRs has fallen well short of the Millennium Development Goal 5. Paradoxically, it is not for a lack of effective, evidence-based interventions that this problem persists. The World Health Organization estimates that at least 88–98% of maternal deaths can be averted with timely access to existing, emergency obstetric interventions [3].

The majority of maternal deaths are clustered around labour, delivery and the 24 hours postpartum [4], [5]. It is estimated that just 5 conditions (postpartum haemorrhage; puerperal sepsis; pre-eclampsia and eclampsia; obstructed or prolonged labour, and complications of unsafe abortion), account for at least 60% of all maternal mortality [6]. Life-threatening situations may develop rapidly and without warning, often in previously uncomplicated pregnancies. It is because of the unpredictable nature of childbirth that emergency obstetric care (EmOC) has been called the ‘keystone in the arch of safe motherhood’ [7].

**Figure 1:**
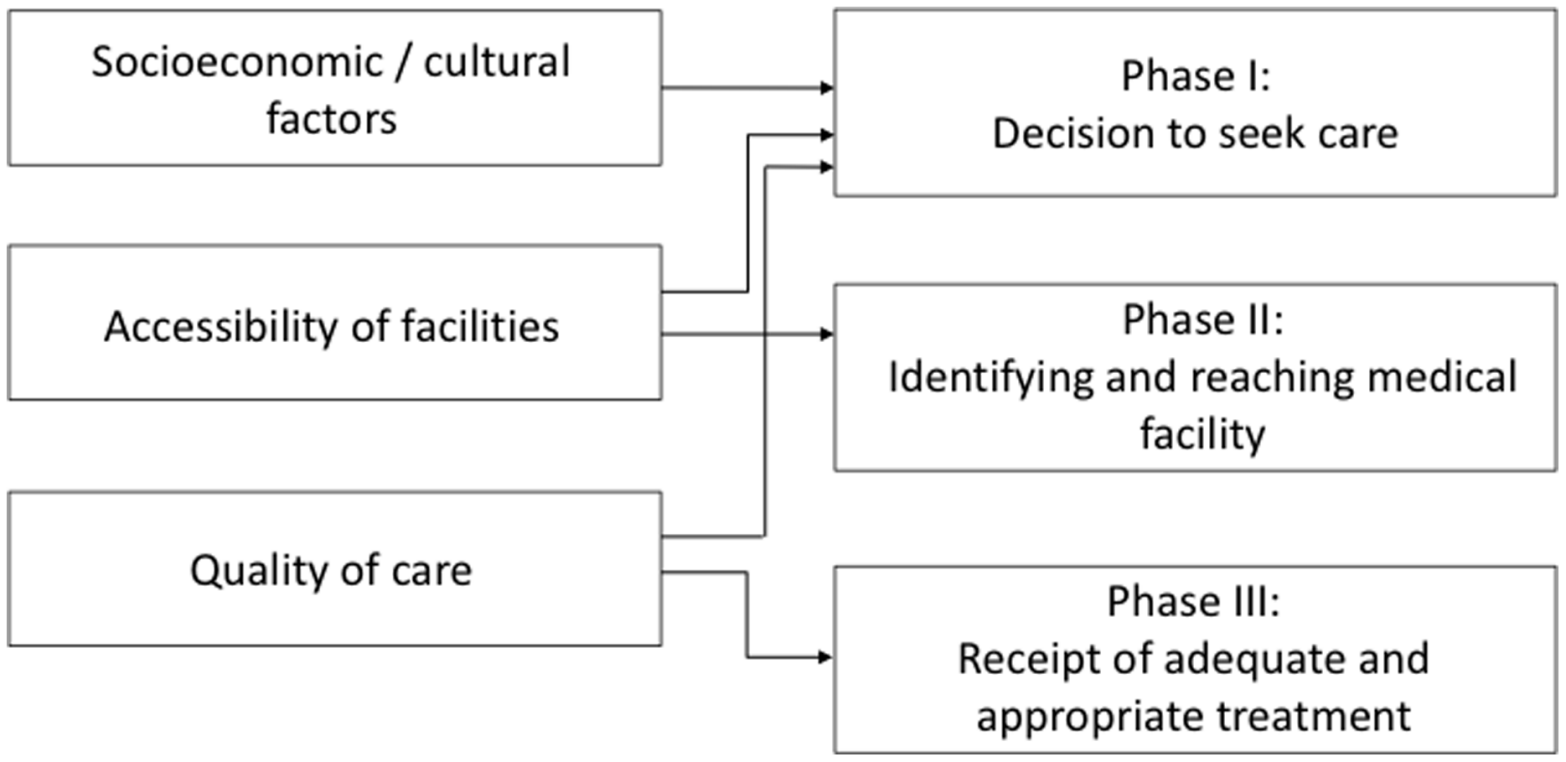
Three Delays Model.

A number of factors can influence a woman’s ability to access effective interventions to treat such complications in the event of an obstetric emergency. In their seminal 1994 paper, Thaddeus and Maine group these into three broad categories using a classic, pathways-based framework [8]. Known as the ‘three delays model’, it has been used extensively in studies of maternal mortality in developing countries (Figure 1).

Since 1994, numerous studies have identified significant Phase I and II ‘demand-side’ barriers (delays in the decision to seek care, and in identifying and reaching a medical facility) that prevent women in developing countries utilising and accessing delivery services. These have been summarised in three systematic reviews [9], [10], [11].

In the wider global health context, there is a growing body of literature on the impact of different health system interventions on health services quality, utilisation and outcomes. The WHO describes six “building blocks” for strengthening health systems: service delivery, health workforce, information, medicines, financing and governance [12]. The emergent field of implementation research has also produced several important reviews on how best to bridge the gap between evidence and practice [13], [14], [15].

Despite these important contributions, no systematic review has dealt comprehensively with the health systems delays that prevent the receipt of timely and appropriate obstetric care once a woman reaches a health facility (Phase III delays). These ‘supply-side’ barriers (for example, lack of adequately trained personnel and difficulties procuring essential drugs) are often major factors contributing to maternal deaths in developing countries.

In this paper we focus on Phase III delays, which, although previously neglected, are likely to be a source of considerable inequity in access to emergency obstetric care. A better understanding of the relative importance of barriers that contribute to Phase III delays could lead to improvements in the quality of obstetric services for women in developing countries.

## Methods

We undertook a systematic review to identify facility-level barriers to the provision of evidence-based, maternal health care in low-income settings, and to identify attempts that have been made to assess the relative importance of these barriers in different settings.

We searched 5 electronic databases (PubMed, CINAHL, CABI Global Health, Global Health Library (Medline) and WHO Publications) from 1994 to 2010 to identify original research articles using a 4-way strategy. We combined search terms (and synonyms) related to: 1) maternal health care (e.g. obstetric care, perinatal care, maternal health services); 2) facility-level (e.g. maternity unit, health facility, Phase III, hospital); 3) barriers (e.g. treatment delay, obstacle, shortage, quality of care), and 4) developing countries (e.g. low-income countries, Africa, Asia, Latin America). The search strategy is available in Appendix S1.

A total of 3,375 papers were retrieved and imported into reference management software (RefWorks, Bethesda, USA). Fifteen additional records were identified from other sources, including a manual search of reference lists of key articles and expert recommendations. After removing duplicates, one investigator (HK) independently screened titles and abstracts to identify candidate articles (n = 53). Articles were shortlisted if the title and abstract indicated that they reported the results of original research studies in the English language using quantitative, qualitative or mixed method approaches. Articles were also required to have been undertaken in developing countries and to report the association between delays at the facility level and maternal mortality or severe morbidity. Articles were also excluded if the research solely examined patient-side or community-level barriers leading to treatment delays, or if only the clinical causes of maternal death were reported. Research published or including data collected before 1994 was excluded, because the ‘three delays’ model was first published in this year, and because the rapid development of maternal health care in the 1990 s reduced the relevance of data from earlier decades.

**Figure 2:**
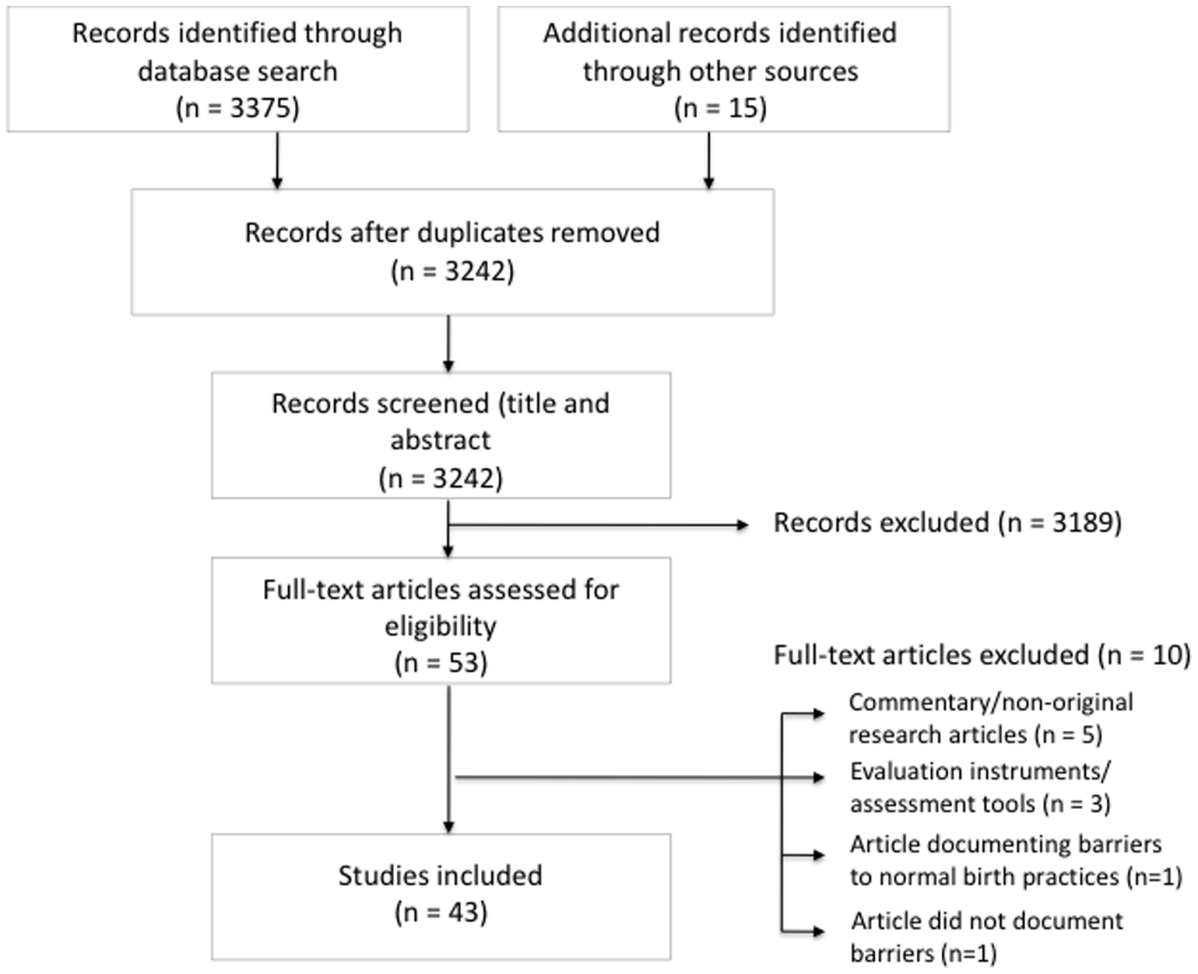
Identification, screening, and inclusion of articles.

Two investigators (HK and AS) then independently reviewed all full-text candidate articles for eligibility. A third investigator (SK) resolved any differences. Ten of the candidate articles were excluded: eight did not report original research (5 were commentary pieces [8], [14], [16], [17], [18]; 3 proposed new evaluation instruments [19], [20], [21]; one dealt with barriers to evidence-based interventions for normal labour [22], and one reported improvements in the quality of maternity care as a result of an intervention without reporting baseline barriers [23]. Forty-three full-text, original research articles were included in the final selection (Figure 2) [24], [25], [26], [27], [28], [29], [30], [31], [32], [33], [34], [35], [36], [37], [38], [39], [40], [41], [42], [43], [44], [45], [46], [47], [48], [49], [50], .

A qualitative approach was used to develop a data extraction framework. Thematic analysis techniques were used to identify and categorise barriers into emerging themes. Starting with a selection of the richest texts, two investigators (HK and AS) recorded all barriers either implicitly or explicitly cited in articles as being responsible for facility-level treatment delays. Barriers that were closely conceptually related were merged. For example, some articles referred to clinical guidelines being out of date or ambiguously worded: these barriers were both classified as ‘inadequate *content* of clinical guidelines’. In contrast, barriers related to poor dissemination or poor enforcement of guidelines on the labour ward were classified as ‘inadequate *dissemination* of clinical guidelines’.

The investigators used the data extraction tool to independently extract the following information from each article: country/ies, type of study (quantitative, qualitative or mixed), methodology (survey, interview, other), sampling strategy, number of facilities/districts covered, interventions covered, and barriers identified, including whether attempts were made to quantify the barriers. A third investigator (SK) resolved any differences.

We identified 32 conceptually unique Phase III barriers, which were categorised into 6 emerging themes (Drugs and equipment; Policy and guidelines; Human resources; Facility infrastructure; Patient-related and Referral-related). Although some authors classify referral barriers as primarily transport-related and therefore Phase II delays, we consider these Phase III barriers since a well-functioning network of primary care integrated with hospital services is a key important component of health service delivery [66]. The frequency with which each barrier was explicitly reported was recorded so as to map trends in the literature (Table 1; Appendix S2). In addition, the reviewers recorded separately any references to factors that they interpreted as being barriers, even if they were not explicitly stated as such by the authors. These were then discussed with the third reviewer (SK) and reported as ‘implicit barriers (Table 1, column 3).

**Table 1:** Frequency of Phase III barriers reported in articles.

	Explicit barriers count(max = 43)	Implicit barriers count(max = 43)	Grand Total
**Human Resources**	40	1	41
Staff shortages	19	7	26
Issues related to quality of training/skills mix	33	4	37
Staff motivation issues	16	3	19
Inadequate supervision	5	5	10
High workload	5	1	6
Authorisation to perform certain tasks	2	2	4
Absenteeism	1	1	2
24-hour availability of staff	8	3	11
Management issues	3	0	3
**Drugs and Equipment**	33	5	38
Cost issues	7	1	8
Inadequate drug supply/logistics problems	26	2	28
Lack of appropriate storage (e.g. fridge/secure cabinet)	2	3	5
Not on essential medicines list/registered for indication	3	0	3
Lack of equipment	18	4	22
Equipment available but not used/faulty	5	1	6
Lack of blood	13	0	13
**Guidelines/Policy**	25	4	29
Inadequate content of clinical guidelines	7	1	8
Inadequate dissemination of clinical guidelines	6	3	9
Poor hospital policy/record keeping	14	0	14
Preference of staff for non-evidence based treatment/s	8	2	10
**Facility Infrastructure**	17	8	25
Lack of beds/ward space	2	2	4
Power/water shortages	8	1	9
Surgical facilities	6	10	16
Transfusion facilities	6	2	8
Laboratory facilities	1	1	2
**Referral**	14	2	16
Distance-related	3	3	6
Road infrastructure	0	2	2
Inadequate emergency transport	12	0	12
Fuel availability	3	0	3
Poor communication	4	1	5
**Patient-side**	13	0	13
Cost-related	8	0	8
Social/cultural/religious	5	1	6

## Results

Of the 43 studies included in the review, 30 were conducted in Africa, 4 in Asia, 4 in Latin America, and 5 in more than one region. The research methods used were quantitative (n = 15), qualitative (n = 9) and mixed (n = 18). Methodologies used to assess Phase III barriers included surveys of healthcare practitioners, in-depth interviews with stakeholders, focus groups, facility-based audits, district-based maternal mortality reviews, and needs assessments based on indicators and signal functions.

### Human Resources

Human resource issues were the most common barriers reported across the literature, mentioned by 41 of the 43 papers. Within this category, it would seem the greatest problems relate in one way or another to training of personnel, as this barrier was cited in 37 articles. There were several accounts of inadequate training resulting in fatalities or near-miss events. One woman, after being admitted with haemorrhagic shock, waited 36 hours before her abdominal pregnancy was diagnosed at laparotomy [24], Several studies reported that educational opportunities for health workers were overwhelmingly deficient due to the absence of continuing education programmes, adequate formal training and a habit of self-learning, as well as poor access to up-to-date educational resources [49], [53] [27], [61]. A Cameroonian midwife taking part in one of the studies remarked: *“I am just happy with these few minutes we have spent today because for me this is my midwifery revision after 34 years”*. She was the midwife in charge of a district hospital maternity unit [53].

The next most cited barriers were staff shortages, referenced in 26 articles, followed by staff motivation issues. Nineteen papers stated that low motivation caused delays. In one hospital in Côte d’Ivoire, the good will of administrative staff was a major factor in whether or not surgical kits could be purchased on credit for critically ill patients, despite the pleas of doctors [24]. Staff were variously identified as overworked and underpaid; [41] misusers of limited health resources for private practice [31]; lacking motivation to change the way they practise [49]. and down-right dishonest [42], [50]. Motivation levels often affected the availability of staff. Both doctors [29] and midwives [27] were found to be more motivated by their private practices, with low wages and poor conditions incentivising staff to work elsewhere, often in cities rather than rural communities [48]. One paper stated: *“the staff had such a low salary that they could not concentrate on their work, but had to spend most of their time in income generating activities”*. Such poor motivation was confirmed by medical officers as an important contributor towards poor patient management [54] and one article recommended that extra incentives should be put in place to retain staff in rural or marginalised areas [48].

Eleven of the articles suggested that 24-hour availability of staff was inadequate. Staff shortages and absenteeism almost certainly contributed towards cases of sub-optimal supervision (ten articles) and high workloads (six articles). Staff shortages were often compounded by managerial issues. In Gondar, Ethiopia, the region’s only obstetrician was often taken from the wards to teach and examine trainees [49], whilst in rural Gambia only one doctor remained on the unit because the other three had been allowed to go on leave at the same time [42].

### Drugs and Equipment

Issues relating to the availability of essential drugs, equipment and blood were cited in 38 articles. Twenty-eight articles referred specifically to inadequate supply and distribution of drugs and equipment, compared with only eight that mentioned cost as a prohibitive factor. Twenty-two articles referred to essential equipment that was lacking altogether (including surgical equipment and vacuum aspirator pumps), as well as very basic equipment such as surgical gloves and cannulae. A further six articles made reference to existing equipment that was either broken or poorly maintained.

Lack of safe blood supplies for transfusion was also a major problem identified in 13 articles. In one Nigerian tertiary hospital over 20% of the maternal deaths were due to delays in acquiring blood [47]. In some cases, blood had to be obtained from hospitals that were several kilometres away [24]; had to be bought by the family at great expense [42], [50], or donated by relatives or friends [43]. Family members were often expected to embark on journeys to obtain blood products [27], sometimes taking several days [42].

Five articles stated that appropriate storage facilities for drugs and blood were not available, and so products became spoiled due to storage at incorrect temperatures. A national survey in Tanzania found that uterotonic drugs (which should be kept refrigerated) were stored at room temperature in 28% of the facilities [46]. During an interview, one Gambian woman recounted, “*My husband managed to buy two bottles of blood for me yesterday. The morning ward staff collected the blood from the lab and put them on top of the ward refrigerator for cooling. The following morning my husband was again told to replace the two bottles as the previously acquired blood was ‘spoiled’ as the nurse put it”* [43].

Finally, in three articles, key drugs were either missing from the country’s ‘essential medicines list’, or not registered for a particular indication. This was a particular issue for magnesium sulphate. For example, in one study, the drug was not licensed for the treatment of pre-eclampsia in 7 out of 13 low-income countries [34]; in Zimbabwe, it had not been registered for use at all [52]. One reason offered was that the low cost of the drug removes any incentive on the part of manufacturers to maximize its use [52].

### Guidelines/policy

Twenty-nine of the papers identified inadequate clinical guidelines or poor policy environment as a factor contributing to sub-standard maternal care in the population being studied. Issues relating to poor policy at the level of the individual facility (for example, bad record keeping) were reported in 14 articles. One of the most common examples of poor policy at the hospital level was the lack of partogram use, which was specifically mentioned in five articles [49], [58], [61], [64], [65]. In one hospital, staff understood the importance of using partograms to monitor the progression of labour but were left to make their own bespoke charts in the medical notes as the hospital did not supply them [49]. Another example was cited in Nairobi, where a policy which dictated that all patients must obtain antenatal cards and pay cash deposits before being eligible for delivery services led to recurrent treatment delays [58].

The inadequate content and dissemination/enforcement of national clinical guidelines was mentioned in eight and nine articles, respectively. The article by Aaserud et al. found that national clinical guidelines for pre-eclampsia were absent in eight out of 13 low and lower-middle income countries, leading to a failure to provide magnesium sulphate when necessary. Those based in Latin America expressed a contrary concern: the over-use of magnesium sulphate in situations in which it was not supported by the latest research, similarly indicating a failure to disseminate best-practice guidelines effectively [34].

Ten articles also reported that staff preferred to use less effective or non-evidence based interventions. These included a preference for expectant rather than active management of the third stage of labour [46]; treatment of eclampsia with diazepam [51] [53]; routine use of enemas [44], and a lack of compliance with recommendations to deliver HIV positive women routinely by Caesarean section [49]. In the article from Cameroon, one medical officer was quoted as saying “*I not only don’t do it [external cephalic version (a recommended practice for breech presentation at term)]; I actively discourage it because it is very dangerous”* [53].

### Facility Infrastructure

Poor hospital infrastructure was identified in 25 articles and the most frequent barrier explicitly reported within this category was a shortage of power and/or water. Nine papers stated that such shortages delay treatment in emergency situations.

Orji et al. concluded that the major factor causing delay to treatment is theatre-related [47]. Indeed we found six studies, which explicitly stated that theatre space and surgical facilities were a problem and a further ten that implied this. Not all hospitals have a theatre but even in those that do, surgical services may be irregular and not accessible 24 hours per day. Delays understandably occur when theatres are already being used, but even when extra theatre space is made available it is not always possible to mobilise staff and so treatment is delayed [24].

Another delay related to transfusion facilities. Many articles stated that the lack of blood delayed treatment, but eight papers found that a number of hospitals did not even have a blood bank. In one paper, only 9% of the district hospitals in Dar Es Salaam had a blood bank [54] and the proportion in another article looking at obstetric care in poor regions of Ghana, India and Kenya found equally poor availability [58]. Bed/ward space and laboratory facilities were less commonly problematic, with only four and two citings respectively.

### Referral-related Factors

It is alarming that even when conditions are identified that require more sophisticated care, providers are sometimes unable to, or worse, unwilling to arrange referrals. Indeed one patient was not referred because of her HIV positive status [41]. Twelve of the 16 articles that highlighted referral-related issues reported that inadequate emergency transport contributed towards maternal mortality. Various reasons were cited such as the ambulance - had broken down and there was no replacement [42]; was being used for an alternative purpose [33], or was under-staffed and ill-equipped [27]. Car ambulances were only available in 7–31% of facilities in three districts of Malawi [28]. Further delays occurred in three of the papers because the ambulance had no fuel [33], [41], [42], meaning at times that the woman’s relatives had to be sent to purchase more [42].

Eight articles mentioned difficult journeys mothers undergo when referred to higher levels of care. Distances between facilities can often be great and the roads themselves poor. One article described the regional hospital as “about 2 hours away on paved roads” [41]. It was also noted that communication between facilities can be sub-standard. Five articles stated that communication was an issue because of non-functioning radios and telephones or a complete lack thereof.

### Patient-side Factors

Thirteen articles reported that patient-side barriers contributed to maternal deaths once women reach a medical facility. Eight articles cited cost-related factors related to compulsory user-fees that hospital staff demand prior to providing treatment/surgery, particularly in relation to emergency Caesarean section. Religious beliefs and negative socio-cultural attitudes towards biomedicine were believed to impede the use of certain medical interventions in six articles. These interventions included HIV testing and administration of peripartum antiretroviral treatment, companionship during labour because of concerns about witchcraft, privacy and gossip [53], and taboos surrounding blood donation that prevented family members from providing or obtaining blood supplies for emergency transfusion [29], [59].

## Discussion

Although there is a relatively large body of literature on health systems barriers in high-income country contexts, the same cannot be said of low-resource settings. By systematically searching 5 electronic databases for original research articles we identified 43 articles that examine Phase III delays in maternal health care in a developing country context. The five most commonly cited barriers were inadequate training/skills mix (86%); drug procurement/logistics problems (65%); staff shortages (60%); lack of equipment (51%) and low staff motivation (44%) (Table 1).

Several of the studies included in this review aimed to assess the relative impact of the three phases of delay on maternal mortality. In some studies, Phase III delays contributed significantly more to maternal mortality than both Phase I and II delays. In a facility-based audit in Tigray, Ethiopia, 88% of the maternal deaths could be attributed to medical failures [30]. In a hospital-based case-control study of maternal mortality in Southern Nigeria, “the most striking difference between the [maternal mortality and control] groups was in the Phase III delays” [25]. In another facility-based maternal death review in Malawi, 20 out of 28 maternal deaths were associated with healthcare worker factors, and a further 6 with administrative failures [62]. Moreover, these findings are not unique to facility-based studies. In a district-based audit in Indonesia, 60% of maternal deaths involved a Phase III delay. [26] In a cohort study of pregnant Haitian women from 10 rural districts, inadequate care at a medical facility was a factor in 7 of the 12 maternal deaths that occurred [41]. Finally, an audit into maternal deaths in a Zimbabwean province found that 87% were avoidable; of these, 57% involved the heath services and 33% patient-related delays [67]. The findings of these studies are supported by the 2005 WHO World Health Report, which estimated that access to good obstetric care could prevent 50–70% of global maternal deaths and substantially reduce the number of women living with sequelae of obstetric complications [68].

The most commonly-identified Phase III barriers identified in this review are related to human resources. Shortages in healthcare personnel are a problem across all health sectors and all levels of training in most of the developing world. The global shortfall is on a massive scale: WHO estimates over 800,000 additional doctors and nurses are required to address current demand [69]. In the field of nursing, there is as much as a hundredfold difference in the nurse to population ratio between some African nations and the United States [70]. Maternal healthcare is no exception: according to a United Nations report, published in 2008, only 47% of deliveries in Sub-Saharan Africa, and 40% in Southern Asia, took place in the presence of a doctor, nurse or midwife [71]. Key reasons include worker shortage, maldistribution, skill mix imbalance, negative work environment, and poor access to in-service training. In many low-income countries, the health workforce is being severely affected by HIV/AIDS, inadequate government investment, and the ‘brain drain’ phenomenon [72]. These barriers will need urgently addressing in order to see further reductions in maternal mortality.

### Strengths and Limitations

There were several limitations to this systematic review. First, a number of rich sources of information about the third delay may have been missed, for example unpublished facility-based audits and confidential enquiries into maternal deaths. Although we sought expert opinion to identify key publications in the grey literature, our search strategy largely identified articles published in peer-reviewed journals. Since many journals have an explicit policy of not publishing audits, a considerable amount of information about Phase III delays may have been missed.

Another problem encountered was related to defining appropriate search terms that would identify all articles relating to Phase III delays. For example, very few medical subject headings (MeSH) were available for treatment delays at the facility level. Instead, free-text search terms had to be developed. Articles about facility-level barriers to the provision of maternal health care do not necessarily refer explicitly to the ‘third delay’ or use other easily predictable terminology. Moreover, there are so many wide-ranging barriers that could impact upon the quality of obstetric care, that it was not possible to anticipate all possibilities in our search strategy. The sheer breadth of the topic means that some important original articles may have been missed. Despite these problems, we believe that although the overview of factors provided in this review is unlikely to be completely exhaustive of the literature on each barrier it is nonetheless a comprehensive review of the categories of barrier that contribute to Phase III delays.

A key challenge related to data extraction was that was that barriers were often poorly defined: for example, many of the articles attributed maternal deaths to the ‘lack of’ a specific drug or resource. With a few exceptions [24], [34], [52], the reasons underlying the poor availability or health commodities were not explored in any depth, making it difficult to classify whether the poor availability was due to cost, supply and distribution, or a combination of factors. Another common problem encountered was the ambiguity of the phrase ‘a shortage of adequately trained staff.’ Is the emphasis here on the number of personnel or the quality of their training, or both? For consistency, in such instances, we decided to record training as an explicit barrier and staff shortage as an implicit barrier.

In some articles, the root cause of poor quality of care was left entirely unexplained. For example, one article cited the case of a woman with diagnosed hand presentation who needed an emergency Caesarean section, yet was left for over 48 hours before having surgery. The authors gave no reasons as to why was she not attended to sooner by staff despite a correct diagnosis [42]. Was it a case of staff lacking the training to know that they should intervene? Was the doctor unavailable? Could the woman simply not afford to pay for the operation? Or were there other factors at work? We chose to document barriers separately that were implied in the text, but not clarified or discussed explicitly (Table 1, column 3).

The quality of the research in many of the articles was methodologically weak. Several papers also failed to explain their methodology adequately, yet made relevant observations that were worthy of comment. Whilst there are clear tools for the assessment of randomized controlled trials and other experimental studies, there is little guidance concerning analysis of the quality of qualitative observational studies. Although several assessment scales and checklists have been used [73], [74], they have not been validated and are inappropriate for non-intervention studies; thus, we included all studies that met the inclusion criteria, regardless of the quality of the quality of the methodology.

The results of this systematic review may be influenced by study bias: that is, the authors of the articles included in the review may have chosen (intentionally or otherwise) to investigate or report certain types of barrier over others, depending on their particular area of interest. Although current knowledge is very limited with regard to the influence of bias on systematic reviews without meta-analyses [75], we acknowledge that the frequency of barriers presented here are not necessarily representative of the “*true frequency*” of barriers in the developing world. The synthesis of results from the reviewed articles (i.e. the frequency counts in Table 1) should be interpreted with some caution, and not extrapolated to represent the true magnitude or relative importance of the various barriers in the developing countries.

The results of the articles are in fact very hard to compare due to the differences in study design, sampling strategy and the analysis techniques employed. Furthermore, context specificity means that synthesis is extremely problematic. As Gabrysch and Campbell highlight in their review of Phase I and II delays [9]:

*“Even if all methods were identical, it would be naïve to expect the effect of, say, distance in Malawi and Peru to be the same, given that infrastructure, transport options, education level, norms around place of delivery and many other factors differ.”*

As this review highlights, a highly complicated web of reasons, many of which are interdependent, can be used explain treatment delays, ranging from behavioural factors such as staff motivation to material factors such as the availability of specific resources. Many of these factors are hard to measure, often go unreported, and are therefore extremely hard to control for to allow context to be taken into account when synthesising results.

Finally, information about which barriers are more or less important to the provision of high quality care was rarely given in the articles reviewed. With the exception of one article [45], studies did not attempt to assess the *magnitude* of barriers in the local context. This type of assessment would be useful for both short- and long-term priority setting purposes. Efforts to incorporate cost-effectiveness into assessments would also be valuable.

### Implications and Recommendations

From an economic perspective alone, global maternal health outcomes would be greatly improved if ways to overcome obstacles to the widespread use of currently available interventions could be found. This review highlights the importance of addressing both supply-and demand-side barriers in any effort to reduce maternal mortality. The studies included document the severity of facility-level failures and serve to highlight that a focus on patient-side delays can sometimes conceal the fact that many health facilities in the developing world are still chronically under-resourced and cannot cope effectively with serious obstetric complications.

Moreover, as Thaddeus and Maine state in their original article, the “three phases of delay rarely operate in isolation…indeed the factors are likely to be interactive and multiplicative. Thus, barriers and poor care encountered at Phase II and III feed back into subsequent decision-making at Phase I” [8]. For this reason, Phase III barriers are likely to influence decisions to seek care and impact not only on maternal mortality at the facility-level, but also on maternal deaths in the community.

The focus in the past few decades on encouraging a shift from home-based to institutional delivery will have been misplaced if efforts to improve the quality of care a woman receives once she comes through the doors of a health facility is not stepped-up. Indicators such as the ‘time from arrival to definitive treatment’ [76] or the ‘percentage of women with obstetric complications treated within two hours at a health facility’ [77] have been proposed. However, few studies reported these outcomes as the data are rarely available from routine medical records [24]. We agree with those who call for the introduction of benchmark indicators that assess the *content and quality* of maternal care, rather than the rates of skilled attendance at birth alone [78].

Simple, replicable tools to assess facility-level barriers are badly needed to assist health managers in identifying facilities that deliver sub-optimal care, and in both making and monitoring the required improvements. No generally accepted methodology exists and this makes comparisons between countries very difficult [19]. We commend efforts by Pitchforth et al. [49] to incorporate social science methodology into these evaluations, as using mixed-method designs may yield more useful results.

To ensure that information on barriers to high quality, effective care contributes to improved responsiveness to emergencies, specific reasons for delays in providing particular interventions must be identified. We have recently completed a large-scale survey of direct maternal healthcare providers in 99 developing countries in an attempt to link specific Phase III barriers to a range of recommended interventions, and to identify locally-appropriate solutions [79].

### Conclusions

This review highlights how a focus on patient-side delays in the decision to seek care sometimes conceals the fact that many health facilities in the developing world are still chronically under-resourced and unable to cope effectively with serious obstetric complications. A wide range of facility-level barriers to EmOC are in operation which result in thousands of avertable deaths worldwide. At present, facility-level barriers are often not reported in sufficient detail in research studies evaluating the quality of maternal healthcare. This makes finding solutions very difficult. We stress the importance of addressing supply-side health systems barriers alongside demand-side factors if further reductions in maternal mortality are to be achieved. The development of simple, replicable tools to assess facility-level barriers should be seen as a priority for future research. The availability of such tools would assist health managers in identifying facilities that deliver sub-optimal care, and in both making and monitoring the required improvements.

## Supporting Information

Appendix S1 **Search Strategy Example (Pubmed).**
(DOCX)

Appendix S2 **Barrier Data Extraction Spreadsheet.**
(DOCX)
