# Supplementary material for: Why Are Women Dying When They Reach Hospital on Time? A Systematic Review of the ‘Third Delay’
Source: PLoS One. 2013 May 21;8(5):e63846. doi: 10.1371/journal.pone.0063846 (PMC3660500; doi:10.1371/journal.pone.0063846)
Supplement: Appendix S1 — Search Strategy Example (Pubmed). (DOCX) [file pone.0063846.s001.docx]

**Appendix S1. Search Strategy Example (Pubmed)**

1. Delivery of Health Care [MH]
2. Health Services Research [MH]
3. Health Care Surveys [MH]
4. Health Policy [MH]
5. Manpower [MH]
6. Equipment and Supplies [MH]
7. #1 OR #2 OR #3 OR #4 OR #5 OR #6
8. barrier* [tiab]
9. delay* [tiab]
10. hurdle* [tiab]
11. prevent* [tiab]
12. shortage* [tiab]
13. lack [tiab]
14. quality[tiab]
15. #8 OR #9 OR #10 OR #11 OR #12 OR #13 OR #14
16. #7 OR #15
17. Maternal Health Services [MH]
18. Postnatal Care [MH]
19. Prenatal Care [MH]
20. Perinatal Care [MH]
21. Maternal Welfare [MH]
22. Maternal Mortality [MH]
23. Delivery, Obstetric [MH]
24. Pregnancy Complications [MH]
25. #17 OR #18 OR #19 OR #20 OR #21 OR #22 OR #23 OR #24
26. maternal health [tiab]
27. matern* care [tiab]
28. intrapartum [tiab]
29. EmOC [tiab]
30. obstetric [tiab]
31. #26 OR #27 OR #28 OR #29 OR #30
32. #25 OR #31
33. Maternal-child Health Centers [MH]
34. Obstetrics and Gynaecology Department [MH]
35. Hospital [MH]
36. Hospitals [MH]
37. Birthing Centers [MH]
38. Rural Health Services [MH]
39. Urban Health Services [MH]
40. Hospitals, rural [MH]
41. Hospitals, urban [MH]
42. Midwifery [MH]
43. Maternal-child Nursing [MH]
44. Obstetrical Nursing [MH]
45. #33 OR #34 OR #35 OR #36 OR #37 OR #38 OR #39 OR #40 OR #41 OR #42 OR #43 OR #44
46. Developing Countries [MH]
47. Africa [MH]
48. Asia [MH]
49. Central America [MH]
50. Latin America [MH]
51. South America [MH]
52. Caribbean Region [MH]
53. #46 OR #47 OR #48 OR #49 OR #50 OR #51 OR #52
54. less-developed countries [tiab]
55. third world [tiab]
56. low-income countries [tiab]
57. developing world [tiab]
58. low-resource [tiab]
59. #54 OR #55 OR #56 OR #57 OR #58
60. #53 OR #59
61. #16 AND #32 AND #45 AND #60
