# Supplementary material for: Why Are Women Dying When They Reach Hospital on Time? A Systematic Review of the ‘Third Delay’
Source: PLoS One. 2013 May 21;8(5):e63846. doi: 10.1371/journal.pone.0063846 (PMC3660500; doi:10.1371/journal.pone.0063846)
Supplement: Appendix S2 — Barrier Data Extraction Spreadsheet. (DOCX) [file pone.0063846.s002.docx]

**Appendix 2. Barrier Data Extraction Spreadsheet**
